# Anemia and its associated factors among women of reproductive Age in Zambia: A multilevel mixed-effects analysis

**DOI:** 10.1371/journal.pone.0330400

**Published:** 2026-02-23

**Authors:** Given Moonga, Florence Mwila, James Muchinga, Mukuka Malasha, Christopher Kalumba, Esau Grecian Mbewe, Garikai Martin Membele, Kumbulani Mabanti

**Affiliations:** 1 Department of Epidemiology and Biostatistics, University of Zambia, Lusaka, Zambia; 2 Center for International Health, Ludwig Maximilian University of Munich, Munich, Germany; 3 School of Business, University of Lusaka, Lusaka, Zambia; 4 Department of Natural Science, Institute of Basic and Biomedical Sciences, Levy Mwanawasa Medical University, Lusaka, Zambia; 5 Department of Geography and Environmental Studies, University of Zambia, Great East Road Campus, Lusaka, Zambia; 6 Department of Health Policy and Management, University of Zambia, Lusaka, Zambia; University of Cape Town Faculty of Science, SOUTH AFRICA

## Abstract

**Background:**

Anemia remains a major global health crisis, affecting over 500 million women of reproductive age, with high burdens in resource-limited regions like Sub-Saharan Africa. Despite ongoing interventions such as iron supplementation programs, 49% of women of reproductive age in Zambia are anemic. Thus, the purpose of this study was to establish the national and subnational prevalence of anemia and identify its determinants among women of reproductive age in Zambia.

**Methods:**

Data were drawn from the 2018 Zambia Demographic and Health Survey (ZDHS), a nationally representative survey employing a stratified two-stage cluster sampling design across 545 enumeration areas. A multilevel mixed-effects logistic regression model was used to identify individual- and community-level factors associated with anemia among women aged 15–49 years (n = 13,055). Four hierarchical models were constructed (null, individual-level, community-level, and full) to assess fixed and random effects, with model selection guided by Akaike Information Criterion (AIC) and Bayesian Information Criterion (BIC) criteria. Spatial analysis was conducted using Quantum Geographic Information System (QGIS), incorporating displaced GPS coordinates in accordance with Demographic Health Survey (DHS) protocols. All analyses applied sampling weights and assessed multicollinearity (Variance Inflation Factor -VIFs < 5).

**Results:**

The national prevalence of anemia among women of reproductive age was 31% (95% Confidence Interval [CI]: 29–33%), with the highest rates observed in Western (38%) and Lusaka (36%) provinces, and the lowest in Central Province (24%). In adjusted analyses, pregnancy (Adjusted Odds Ratio [AOR] = 1.76; 95% CI: 1.52–2.03), Human Immunodeficiency Virus (HIV) positivity (AOR = 2.21; 95% CI: 1.97–2.49), and breastfeeding (AOR = 1.15; 95% CI: 1.02–1.30) were significantly associated with increased odds of anemia. Conversely, being married (AOR = 0.78; 95% CI: 0.68–0.90) and age 25–29 years (AOR = 0.84; 95% CI: 0.71–0.97) were protective. Spatial mapping identified Western Province as a high-burden hotspot. Community-level variance was notable (Intraclass Correlation Coefficient [ICC] = 6%, Median Odds Ratio [MOR] = 1.52), with 5% residual clustering persisting after adjusting for both individual and contextual factors, suggesting the influence of unmeasured ecological determinants.

**Conclusion:**

Anemia remains a significant public health issue among Zambian women of reproductive age, shaped by both individual- and community-level factors. These findings highlight the need for integrated, targeted interventions focusing on high-risk groups in high-prevalence areas. Strengthening clinical services and implementing community-based strategies to address healthcare access and environmental determinants are essential to reducing the burden of anemia in Zambia.

## Introduction

Anemia is a condition characterized by a reduction in the number of red blood cells and the concentration of hemoglobin (Hb), which reduces the blood’s ability to carry adequate oxygen to meet the body’s physiological need [1]. The World Health Organization (WHO) defines anemia in non-pregnant women as a hemoglobin (Hb) concentration of less than 12 g/dL and and below 11 g/dL in pregnant women [1]. Anemia is a significant global public health concern, affecting over 500 million women of reproductive age (WRA) worldwide [2]. WHO estimates that 37% of pregnant women and 30% of women aged 15–49 years worldwide are anemic [1].

The burden of anemia is disproportionately high in low-and middle-income countries (LMICs) and has negative impacts on maternal and child health which includes low birth weight, preterm birth, poor cognitive development perinatal and neonatal mortality, maternal morbidity and mortality [3, 2]. Additionally, anemia leads to reduced productivity, cognitive impairment, and increased susceptibility to infections due to compromised immune function. It also affects nervous the system, respiratory and circulatory system, skin mucous membrane, digestive system, endocrine system [4, 5]. Africa and India bear the highest burden of anemia globally, with nearly half of all women affected. In these regions, anemia contributes to 40% of maternal deaths [6]. The greatest risk of anemia among women is seen in Sub-Saharan Africa (SSA), where 46% of pregnant women, and 39% of women of reproductive age are affected [7]. In Eastern Africa, anemia prevalence among women aged 15–49 years varies significantly, ranging from 19.2% in Rwanda to 49% in Zambia where an estimated 36.2% of pregnant women are affected. This high burden of maternal anemia represents a significant public health concern in a country already challenged by elevated maternal mortality [8].

There are several factors associated with anemia in pregnancy; including micronutrient deficiencies such as iron, folate, and vitamins A and B12, as well as infectious diseases like HIV, tuberculosis, malaria and hookworm. Among these factors, iron deficiency accounts for approximately 50% of anemia cases[10]. Iron deficiency is common among women of reproductive age, largely due to increased iron requirements during pregnancy and lactation, menstrual blood loss, and inadequate nutritional intake throughout the reproductive cycle [9].

Efforts to reduce anemia among women of reproductive age are being made in most countries including Zambia through iron supplementation and health education on nutrition. However, existing patterns of social inequalities in anemia over time among women of reproductive age are not well studied in most countries in the Sub-Saharan Region including Zambia. Existing disparities across places of residence and geographical areas are not well known [6, 10]. For instance, a study in India revealed that anemia prevalence has increased significantly over a recent 7-year period, despite socioeconomic inequalities (by wealth, education and residence), having decreased over time [11].

The mixed-effects multivariable multilevel analysis becomes suitable to assess variation of anemia prevalence most studies use random effects to capture group specific variation in the study how they influence occurrence of anemia and these factors include community level and individual factors [12]. Evidence from prior research supports this approach. For example Teshale et al., (2020) reported a higher prevalence of anemia among rural women in Ethiopia (25.1%), compared with their urban counterparts (17.5%). Their findings further demonstrated that community factors, including, low household wealth, were strongly associated with anemia in rural regions, underscoring the combined influence of geographic and socioeconomic factors on anemia prevalence [13].

Hence, this study examined patterns of anemia prevalence among women of reproductive age in Zambia; identified key determinants and assessed regional disparities. The findings provide evidence that is relevant to efforts aimed at achieving the World Health Organization’s global target of a 50% reduction in anemia among women of reproductive age by 2030.

## Methods

### Study design and setting

This study involved a secondary analysis of data from the 2018 Zambia Demographic and Health Survey (ZDHS). The ZDHS is a nationally representative survey conducted by the Zambia Statistics Agency (ZamStats) in collaboration with the Ministry of Health (MOH) and ICF International, using a stratified two-stage cluster sampling design. Comprehensive details on the sampling methodology design, data collection procedures, and survey instrument are provided in the ZDHS final report [14]. The primary objective of the 2018 ZDHS was to collect up-to-date and comprehensive data on vital demographic and health indicators to inform national policy and planning.

### Sampling and data measurement of ZDHS

The ZDHS employed a two-stage sampling design to ensure representativeness at national, provincial, and urban–rural levels. In the first stage, enumeration areas (EAs) were selected as clusters using probability proportional to size within each stratum, with a total of 545clusters included. In the second stage, a household listing was conducted within each cluster, identifying an average of 133 households per cluster. From these, 25 households per cluster were systematically sampled, yielding a total of 13,625 households. The detailed methodology of the ZDHS has been well-documented in prior publications [14].

Anemia testing was done on all women of reproductive age (15–49 years old) from whom consent was obtained [14]. The missing data constituted only 4% and was, therefore, negligible since 96% of the selected women for the interviews responded.

### Explanatory variables (Risk Factors)

The study incorporated both individual-level and community-level variables available in the ZDHS dataset. Previous studies have attributed the prevalence of anemia to both individual and community-level factors.

Individual-level factors encompassed demographic and health-related characteristics such as age, religion, marital status, educational attainment, wealth index, and gravidity. Community-level factors included variables such as residence type (urban or rural), geographic region, water source, and type of toilet facilities, which reflect populations living under similar environmental or infrastructural conditions. The classification of variables as individual- or community-level was guided by the assumption of independence between levels.

This study used secondary data that containing no personal identifier; therefore, the requirement for individual informed consent was waived by the University of Zambia Biomedical Research Ethics Committee (UNZABREC), which approved the study (Approval Number: 6300−2025).

### Statistical analysis

Statistical analysis was conducted using Stata 17 to generate descriptive statistics and address the research questions under investigation. Frequencies and percentages were used to summarize both individual- and community-level variables. Given that some regions with smaller populations were oversampled while others with larger populations were underrepresented, weighted frequencies and percentages were calculated to account for population size differences across regions. The detailed weighting methodology is outlined in the ZDHS 2018 report [14].

To identify factors associated with anemia among women, bivariate logistic regression analyses were first conducted to select variables for inclusion in both the multivariate standard logistic regression and mixed-effects multilevel logistic regression models. Missing observations for the anemia and HIV variables, as well as inconclusive responses in the HIV variable, were excluded, reducing the sample from 13,625–13,055 observations.

To select between the multivariate standard logistic regression and the mixed-effects multivariable multilevel logistic regression model, a log-likelihood ratio test was conducted. A model with a higher log-likelihood value was preferred [15].

### Standard logistic model


$$\begin{array}{c} ogit\left(\pi_{j}\right)=\;\beta_{\text{0}}+\;\beta_{\text{1}}Respondent\_age+\;\beta_{\text{2}}Marital\_status+\;\beta_{\text{3}}Sex\_Hh+\;\beta_{\text{4}}Currently\_preg\\ +\;\beta_{\text{5}}Currently\_breast+\;\beta_{\text{6}}Preceding\_\text{5}yrs+\;\beta_{\text{7}}HIV\_test+\;\beta_{\text{8}}Religion\\ +\;\beta_{\text{9}}Province+\;\mu \end{array}$$ (1)

### Mixed-effects multilevel model

A mixed effects multilevel model is a standard logistic regression model, amended to have random effects for each EA. Defining $\pi_{ij}=\text{Pr }(Anemia_{status}=\text{1})$, we have


$$logit\left(\pi_{ij}\right)=\;\beta_{\text{0}}+\;\beta_{\text{1}}{Respondent\_age}_{ij}+\;\beta_{\text{2}}{Marital\_status}_{ij}+\;\beta_{\text{3}}{Sex\_Hh}_{ij}+\;\beta_{\text{4}}{Currently\_preg}_{ij}\;+\;\beta_{\text{5}}{Currently\_breast}_{ij}+\;\beta_{\text{6}}{Preceding\_\text{5}yrs}_{ij}+\;\beta_{\text{7}}{HIV\_test}_{ij}+\;\beta_{\text{8}}{Religion}_{ij}\;+\;\beta_{\text{9}}{Province}_{ij}+\;\mu_{j}$$ (2)

for *j* = 1,..., 545 EAs, with *i* = 1,..., $n_{j}$ women in EA *j*.

### Model selection

A log-likelihood ratio test was conducted to select between the standard logistic regression and the mixed-effects multilevel logistic regression model as shown in Table 1. As a rule of thumb can be seen that a model with a higher log-likelihood value is preferred [15].

**Table 1:** Standard logistic and Mixed effects multilevel regressions.

Model	Log-likelihood Ratio
Standard logistic regression	−7811
**Mixed effects multilevel regression**	**−7757**

### Mixed-effects logistic regressions

We specified 4 distinct models, aiming to identify the risk factors at different levels, model 1 included no predictors to assess community-level variation in anemia prevalence; model 2 added individual-level factors; model 3 included community-level factors; and Model 4 combined both individual- and community-level factors. In more detail, the differences between the models are summarized in Table 2.

**Table 2:** Specification of estimated models.

Model term	Model 1	Model 2	Model 3	Model 4
$\beta_{\text{0}}$	y^a^	y	y	y
$\beta_{\text{1}}Respondent\_age$	n^b^	y	n	y
$\beta_{\text{2}}Marital\_status$	n	y	n	y
$\beta_{\text{3}}Sex\_Hh$	n	y	n	y
$\beta_{\text{4}}Currently\_preg$	n	y	n	y
$\beta_{\text{5}}Currently\_breast$	n	y	n	y
$\beta_{\text{6}}Preceding\_\text{5}yrs$	n	y	n	y
$\beta_{\text{7}}HIV\_test$	n	y	n	y
$\beta_{\text{8}}Religion$	n	y	n	y
$\beta_{\text{9}}Province$	n	n	y	y

^a^Yes (y) and ^b^No (n).

The fixed-effects results were expressed as odds ratios (ORs) with 95% confidence intervals (CIs), while adjusted odds ratios (AORs) with 95% CIs were calculated to identify independent factors associated with anemia. Statistical significance was determined using a p-value threshold of <0.05. To assess multicollinearity, variance inflation factors (VIFs) were computed, with variables considered to have low collinearity if their VIF values were below [16].

Random effects, capturing variations between clusters, were assessed using the Intra-Cluster Correlation Coefficient (ICC), Percentage Change in Variance (PCV), and Median Odds Ratio (MOR). The ICC quantified the proportion of total variability attributable to clustering effects, while the MOR converted unexplained cluster-level variance into the OR scale, facilitating the interpretation of heterogeneity. The PCV measured the proportion of total variance explained by individual- and community-level factors. These measures collectively informed the suitability of the model specification. These measures were computed using the following formulas:


$$ICC=\;\frac{V}{V\;+\;\frac{\pi^{\text{2}}}{\text{3}}}$$ (3)

$$MOR=exp\sqrt{\text{2}\;\times V\;\times \text{0}.\text{6}\text{7}\text{4}\text{5}\;}\;\sim \text{ exp}(\text{0}.\text{95}\sqrt{V)}$$ (4)

$$PCV=\;\frac{V_{A}\;+\;V_{B}}{V_{B}}\;\times \text{1}\text{0}\text{0}$$ (5)

Where V represents the estimated variance of clusters, $V_{A}$ is the variance in the initial model, and $V_{B}$ is the variance in subsequent models with additional terms. The multi-level analysis was well suited for our study given the correlated nature of our data. It evaluates how variables at different levels influence the dependent variable while addressing biases introduced by clustering [17]. By producing corrected standard errors, confidence intervals, and significance tests, multilevel models yield reliable parameter estimates [18].

The results of the fourth model, selected based on the AIC and BIC, indicated that age group, marital status, sex of the household head, pregnancy status, breastfeeding status, number of children born in the preceding five years, HIV test results, residence, and religion were significantly associated with anemia among women.

### Spatial mapping

The ZDHS data is georeferenced, enabling spatial analysis to be carried out. To maintain the confidentiality of respondents, ZDHS has a georeferenced data release policy, which provides two levels of protection. First, data from the same enumeration area (EA) are aggregated to a single-point coordinate. Second, a GPS coordinate displacement process is applied, which masks the true locations. Urban clusters were displaced a distance of up to 2 km (0–2 km), rural clusters up to 5 km (0–5 km), and an additional randomly selected 1% of rural clusters are displaced up to ten kilometers (0–10 km) [14].

A spatial analysis was conducted to examine the geographic distribution of anemia prevalence among women of reproductive age (WRA) in Zambia using geospatial data from the 2018 Zambia Demographic and Health Survey (ZDHS). A shapefile containing Zambia’s administrative boundaries with province-level anemia prevalence estimates (percentages) was obtained from the ZDHS website. The geospatial data were imported into QGIS for spatial processing and visualization. Anemia prevalence was classified into seven categories and displayed using a graduated color scale, with yellow representing the lowest prevalence and red the highest prevalence. Provincial labels were added to enhance interpretability. The resulting map illustrates spatial disparities in anemia prevalence across Zambia’s provinces.

## Results

### Sociodemographic characteristic

Table 3 presents the descriptive statistics of the study participants. The analysis included data on a total of 13,055 women from 545 clusters across 10 provinces. The median age (IQR) of the participants was 27 (20 –36) years. Over half (56%) of the women were married, and 8% had no formal education. About half (54%) of the women in the study were rural residents, and 71% identified as protestant Christians. About 8% of the women were pregnant at the time of the survey, while 23% were breastfeeding and 14% were HIV positive. Furthermore, nearly three-quarters (73%) of the households were male-headed, 72% had access to improved water sources while 57% had unimproved toilet facilities.

**Table 3:** Descriptive statistics and prevalence of anemia among study participants (n = 13,055).

Covariates	Weighted Frequency	Weighted percent	Anemic	Non-Anemic	Weighted Frequency of Anemia	*P-value
**Age Groups (years)**						
15-19	2878	22%	958	1920	33% (31 –36)	0.005
20-24	2626	20%	741	1885	28% (26 –31)	
25-29	2120	16%	611	1509	29% (26 –32)	
30-39	3380	26%	1058	2321	31% (29 –34)	
40-49	2051	16%	679	1373	33% (31 –36)	
**Marital Status**						
Single	4061	31%	1377	2684	34% (32 –36)	<0.001
Married	7281	56%	2060	5220	28% (27 –30)	
Divorced	1713	13%	610	1104	36% (33 –38)	
**Education Level**						
Higher education	698	5%	246	451	35% (31 –40)	0.323
Secondary	5532	42%	1721	3811	31% (29 –34)	
Primary	5831	45%	1773	4059	30% (29 –32)	
No education	994	8%	307	687	31% (27 –35)	
**Sex of household head**						
Male	9514	73%	2840	6674	30% (28 –31)	<0.001
Female	3541	27%	1207	2334	34% (32 –36)	
**Residence**						
Urban	6013	46%	1931	4082	32% (30 –35)	0.163
Rural	7042	54%	2116	4926	30% (29 –32)	
**Region**						
Central	1133	9%	269	864	24% (21 –27)	<0.001
Copperbelt	2042	16%	600	1442	29% (26 –33)	
Eastern	1541	12%	425	1116	28% (24 –31)	
Luapula	967	7%	286	681	30% (27 –33)	
Lusaka	2658	20%	945	1713	36% (32 –39)	
Muchinga	742	6%	205	537	28% (22 –33)	
Northen	1039	8%	289	749	28% (24 –32)	
North western	690	5%	224	467	32% (28 –37)	
Southern	1486	11%	520	966	35% (31 –40)	
Western	757	6%	284	473	38% (34 –41)	
**Religion**						
Catholic	2245	17%	682	1563	30% (28 –33)	<0.001
Protestant	10587	81%	3273	7314	31% (29 –32)	
Muslim	61	1%	13	48	21% (13 –33)	
Other	162	1%	79	83	49% (39 –58)	
**Wealth Index**						
Poorest	2334	18%	702	1632	30% (28 –32)	0.548
Poorer	2307	18%	704	1602	31% (28 –33)	
Middle	2383	18%	725	1658	30% (28 –33)	
Richer	2906	22%	894	2013	31% (28 –34)	
Richest	3125	24%	1022	2103	33% (30 –36)	
**Currently Pregnant**						
Yes	1074	8%	442	632	41% (36 –46)	<0.001
No	11981	92%	3605	8376	30% (29 –31)	
**Currently Breastfeeding**						
Yes	2985	23%	822	2163	28% (26 –30)	<0.001
No	10070	77%	3225	6845	32% (30 –34)	
**Gravidity of women (Children ever born)**						
0	3191	24%	1106	2085	35% (32 –37)	<0.001
1-3	5472	42%	1657	3815	30% (28 –32)	
4+	4392	34%	1284	3108	29% (28 –31)	
**Children ever born in the preceding 5 years**						
0	6013	46%	2053	3960	29% (26 –31)	<0.001
1	4820	37%	1381	3439	28% (25 –30)	
2+	2222	17%	613	1609	35% (32 –37)	
**Smoking**						
Yes	346	3%	89	257	26% (21 –31)	0.059
No	12709	97%	3958	8751	31% (30 –33)	
**HIV test**						
Positive	1863	14%	843	1020	45% (42–49)	<0.001
Negative	11192	85%	3204	7988	29% (27 –30)	
**Getting medical help for self: distance to health facility**						
Big problem	3794	29%	1189	2606	31% (30 –33)	0.715
Not a big problem	9261	71%	2858	6402	31% (29 –33)	
**Have mosquito bed net for sleeping**						
Yes	10601	81%	3236	7365	33% (30 –36)	0.082
No	2454	19%	811	1643	31% (30 –33)	
**Type of water source**						
Improved	9425	72%	2954	6571	31% (30 –33)	0.503
Unimproved	3291	25%	984	2307	30% (28 –32)	
Other	339	3%	109	230	32% (26 –39)	
**Type of toilet**						
Improved	4211	32%	1317	2895	31% (29 –34)	0.847
Unimproved	7425	57%	2275	5149	31% (29 –32)	
Open	1095	8%	353	742	32% (29 –36)	
Others	324	3%	102	222	31% (25 –38)	

*p-values generated from Pearson chi2-test, Wealth Index: Categorized into quintiles based on household assets and living conditions: Poorest, Poorer, Middle, Richer, and Richest, Getting medical help for self: Indicates whether obtaining medical care for the respondent is perceived as a big problem or not a big problem, HIV: Human Immunodeficiency Virus.

Table 3 shows the variation of anemia across different Socio-demographic and health-related characteristics among study participants. The estimated anemia prevalence among women of reproductive age was 31%. Significant regional variations were observed, Lusaka and Western had the highest prevalence (36% and 38%, respectively), while Central province had the lowest (24%). Adolescent women (aged 15–19) years and older women (40–49 years) had the highest and equal anemia prevalence (33%). Single women exhibited higher anemia prevalence (34%) compared to married women (28%). The prevalence of anemia among pregnant women was higher (41%) compared to the women who were not pregnant (30%).

Factors such as marital status, HIV positivity, current pregnancy, current breastfeeding, and gravidity were significantly associated with anemia prevalence (p = 0.001), whereas access to medical care, place of residence, and sanitation showed no significant association. Gravidity was excluded from the multivariate standard logistic regression and mixed-effects multilevel logistic regression models to minimize multicollinearity, given its strong correlation with age and marital status in the study population.

**Fig 1:**
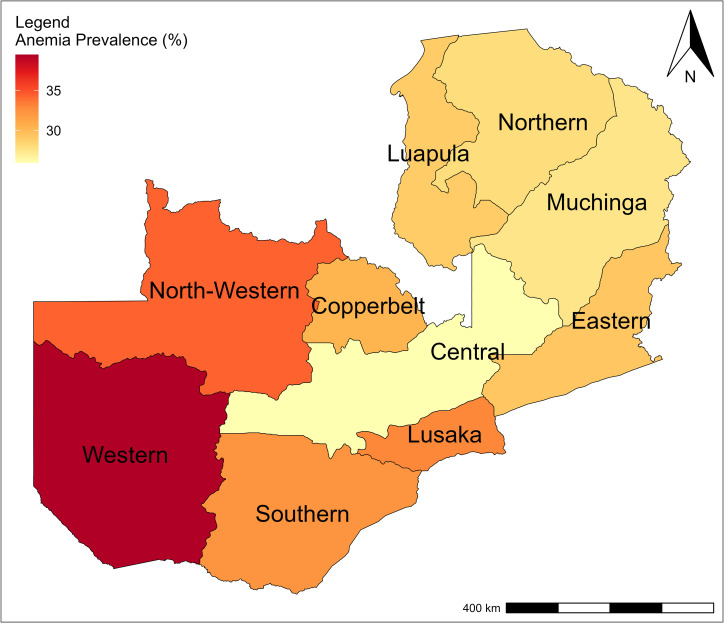
Map of Zambia showing the prevalence of anemia at provincial level. The base layer of the map was obtained from the Database of Global Administrative Areas (GADM) version 4.1 (https://gadmorg/download_country.html). GADM data is freely available for academic and non-commercial use and is compatible with the CC BY 4.0 license. Anemia prevalence data were obtained from the 2018 Zambia Demographic and Health Survey.

The spatial distribution of anemia prevalence in Zambia is shown on a map using a sequential color gradient, with lighter shades indicating lower prevalence and darker shades indicating higher prevalence. The lowest prevalence is observed in Central Province and the highest in Western Province as shown in Fig 1.

### Mixed effect

Table 4 summarizes the multilevel mixed-effects logistic regression estimates for determinants of anemia among women of reproductive age. Women aged 25–29 years old were 16% less likely to be anemic compared with women in the youngest age group (15–19 years old) (AOR = 0.84; 95% CI 0.71 to 0.97). The married women were 0.78 times less likely to be anemic than women who never married (AOR = 0.78; 95% CI 0.68 to 0.90). The odds of anemia were higher in women who were pregnant (AOR = 1.76; 95% CI 1.52 to 2.03) compared with those who were not pregnant. Women who were currently breastfeeding were 15% (AOR = 1.15; 95% CI 1.02 to 1.30) more likely to be anemic. Women who gave birth to one child in the preceding 5 years of the survey were at low risk of having anemia (AOR = 0.84; 95% CI 0.76 to 0.94). In this study, women who were HIV positive had a twofold increased odds of having anemia compared with women classified as HIV negative (AOR = 2.21; 95% CI (1.97 to 2.49). Higher odds of anemia were observed in Copperbelt(AOR = 1.33; 95% CI 1.06 to 1.68), Eastern (AOR = 1.27; 95% CI 1.01 to 1.61), Lusaka (AOR = 1.3; 95% CI 1.03 to 1.66), Luapula (AOR = 1.52; 95% CI 1.21 to 1.90), Northern (AOR = 1.31; 95% CI 1.03 to 1.67), Northwestern (AOR = 1.74; 95% CI 1.36 to 2.22), Southern(AOR = 1.52; 95% CI 1.20 to 1.91) and Western provinces (AOR = 1.99; 95% CI 1.56 to 2.54) compared with Central province.

**Table 4:** Multilevel mixed-effects logistic regression of factors associated with anemia among women of reproductive age.

Variables	Model 1	Model 2 Individual-level factors OR (95% CI)	Model 3 Community-level factors OR (95% CI)	Model 4 Individual + community-level factors OR (95% CI)
**Age Groups**				
15-19		1		1
20-24		0.89 (0.78 to 1.02) *		0.88 (0.77 to 1.01) *
25-29		0.84 (0.72 to 0.99) **		0.83 (0.71 to 0.97) **
30-39		0.97 0.84 to 1.13)		0.95 (0.82 to 1.11)
40-49		1.04 (0.88 to 1.23)		1.02 (0.86 to 1.20)
**Marital Status**				
Single		1		1
Married		0.76 (0.66 to 0.87) ***		0.78 (0.68 to 0.90) ***
Divorced		0.89 (0.76 to 1.04)		0.91 (0.77 to 1.07)
**Sex of household head**				
Male		1		1
Female		1.04 (0.94 to 1.16)		1.03 (0.93 to 1.14)
**Currently Pregnant**				
No		1		1
Yes		1.77 (1.53 to 2.04) ***		1.76 (1.52 to 2.03) ***
**Currently Breastfeeding**				
No		1		1
Yes		1.16 (1.03 to 1.31) **		1.15 (1.02 to 1.30) **
**Children ever born in the preceding 5 years**				
0		1		1
1		0.85 (0.76 to 0.95) ***		0.84 (0.76 to 0.94) ***
2+		0.91 (0.78 to 1.06)		0.991 (0.78 to 1.06)
**HIV test**				
Negative		1		1
Positive		2.22 (1.98 to 2.50) ***		2.21 (1.97 to 2.49) ***
**Religion**				
Catholic		1		1
Protestant		1.02 (0.92 to 1.14)		1.01 (0.90 to 1.12)
Other		1.30 (0.93 to 1.82)		1.27 (0.91 to 1.78)
**Region**				
Central			1	1
Copperbelt			1.35 (1.08 to 1.69) ***	1.33 (1.06 to 1.68) **
Eastern			1.20 (0.96 to 1.50)	1.27 (1.01 to 1.61) **
Luapula			1.25 (0.99 to 1.57) *	1.31 (1.03 to1.66) **
Lusaka			1.52 (1.22 to 1.89) ***	1.52 (1.21 to 1.90) ***
Muchinga			1.05 (0.82 to 1.34)	1.15 (0.89 to 1.47)
Northen			1.20 (0.95 to 1.51)	1.31 (1.03 to 1.67) **
North western			1.62 (1.28 to 2.06) ***	1.74 (1.36 to 2.22) ***
Southern			1.48 (1.18 to 1.86) ***	1.52 (1.20 to 1.91) ***
Western			1.99 (1.57 to 2.52) ***	1.99 (1.56 to 2.54) ***
**Random effects**				
Community level variance (SE)	0.19 (0.03)	0.20 (0.03)	0.15 (0.02)	0.16 (0.02)
P value	<0.000	<0.000	<0.000	<0.000
DIC (−2log likelihood)	−7931.79	−7778.51	−7905.97	−7756.62
ICC (%)	5.55	5.83	4.50	4.90
Explained variation – PCV (%)	Reference	−5.17	24.78	13.92
MOR	1.52	1.54	1.45	1.48

Note: *** p < 0.01, ** p < 0.05, * p < 0.1. DIC, Deviance Information Criterion; ICC, Intra-Cluster Correlation Coefficient; MOR, Median OR; PCV, Percentage Change in Variance, OR: Odds Ratio, CI: Confidence Interval, SE: Standard Error.

### Random effects

As shown in Table 4, the random-effects model demonstrates significant variation in anemia prevalence across communities (p < 0.001). In other words, the anemia prevalence rate was not similarly distributed across the communities. About 6% of the variance in the odds of anemia in women could be attributed to community-level factors, as calculated by the ICC based on estimated intercept component variance. After adjusting for individual-level and community-level factors, the variation in anemia across communities remained statistically significant. About 5% of the odds of anemia variation across communities was observed in the full model (model 4). In the null model, the MOR for anemia was 1.52, indicating that there was a variance between communities (clustering) (1.52 times larger than the reference (MOR = 1). When both individual and community factors were included in the model, the unexplained community variation in anemia decreased to MOR of 1.48. This indicates that in the full model the effects of clustering are still statistically significant when we considered both individual and community factors.

The fit of the models are compared by relying on Akaike Information Criterion (AIC) and the Bayesian Information Criterion (BIC) and are summarised in Table 5. As can be seen that the model with the lowest value describes the data best [19].

**Table 5:** Estimation results: AIC and BIC.

Model	AIC	BIC
Model 1	15868	15868
Model 2	15586	15683
Model 3	15838	15942
**Model 4**	**15561**	**15755**

## Discussion

In this study approximately 31% WRA were anemic a level that, according to World Health Organization (WHO) classification, constitutes a moderate public health burden in Zambia. The study also assessed the spatial distribution and key determinants of anemia, highlighting geographic and socioeconomic inequalities that shape anemia risk in the country.

The prevalence of anemia among women of reproductive age in Zambia exceeds the global average of 29.9% [19] and remains higher than estimates reported in several neighboring and comparable countries, including Ethiopia (21.7% among women aged 15–24) and Rwanda (19.2% among 6,680 women aged 15–49) reflecting heightened vulnerability among Zambian women due to biological factors, such as recurrent blood loss and gestational demands, as well as socio-environmental determinants, including food insecurity and limited access to healthcare [20], Nonetheless, Zambia’s anemia emains lower than in several other sub-Saharan African countries, including the Democratic Republic of the Congo (42.4%), Burundi (38.5%), South Sudan (35.6%), the Central African Republic (46.8%), Tanzania (38.9%), Uganda (32.8%), Congo-Brazzaville (48.8%), and Angola (44.5%) [19], underscoring the need for targeted interventions to sustain progress and reduce disparities.

Spatial analysis revealed significant geographic variation in anemia prevalence, which may reflect provincial-level differences in dietary patterns, communicable disease burden, and healthcare access. Additionally, environmental determinants such as access to clean water and sanitation were also noted to influence anemia risk through increased exposure to soil-transmitted helminths [21]. Western province recorded the highest anemia prevalence (38%), followed closely by Lusaka province (36%). The elevated burden in Western province may stem from widespread poverty and reliance on subsistence farming, which limits access to iron-rich and diverse diets. In Lusaka, a study attributed anemia risk to poor vegetable intake and low socioeconomic status, particularly in informal urban settlements [22]. Consistent with previous findings in LMICs, this study also found that women residing in rural areas were more likely to be anemic than their urban counterparts. Contributing factors may include limited access to nutritious foods, lower socioeconomic conditions, and poor sanitation, all of which elevate disease exposure and contribute to increased anemia risk [23].

The multilevel mixed effects model indicated that both individual- and community-level factors accounted for approximately 14% of the variation in anemia prevalence. Age was a significant predictor; women aged 25–29 years had lower odds of anemia than those aged 15–19 years, consistent with findings from Ethiopia and Benin [21]. This could reflect lower fertility rates and better health-seeking behaviors among women in their mid-20s, including higher adherence to antenatal supplementation [4]. However, contradictory findings from Burkina Faso suggest an increased risk of severe anemia in this age group, potentially due to ongoing physiological development and menstrual iron loss compounded by inadequate dietary intake [24]. Marital status was also found to be a protective factor against anemia. Married women were less likely to be anemic than never-married women, likely due to increased access to financial, emotional, and nutritional support within marital households [25]. Such support may improve dietary adequacy and access to healthcare services, indirectly reducing anemia risk.

Pregnancy was positively associated with anemia, echoing evidence from Sub-Saharan Africa and LMICs, including Ethiopia, Mali, and Gambia [26]. The physiological changes that occur during pregnancy including blood volume expansion, fetal growth, and placental development elevate iron requirements, which may not be met through diet alone, particularly in resource-constrained settings [25]. Nonetheless, studies from Rwanda have reported no significant association between pregnancy and anemia, underscoring the importance of country-specific contextual factors [27]. Breastfeeding was similarly associated with a higher risk of anemia, consistent with studies from Ethiopia, India, and the Democratic Republic of Congo [19]. The increased nutrient demands during lactation, particularly for vitamins such as thiamine, riboflavin, B6, B12, A, and iodine, may not be adequately met among Zambian women, especially in rural areas where dietary diversity is limited [19]. These deficiencies can compromise hemoglobin synthesis and maternal iron stores during the postpartum period.

Women who had given birth to one child in the past five years were less likely to be anemic than those who had not given birth during that period. This finding aligns with evidence from Zambia and East Africa [28] and may reflect increased engagement with maternal health services and heightened health awareness during and after pregnancy. However, it contrasts with evidence from Mali and Ethiopia, where high gravidity and frequent births were associated with elevated anemia risk due to repeated blood loss, nutritional depletion, and parasitic infection [29]. These contrasting results suggest that the relationship between parity and anemia is complex and context-dependent. Lastly, HIV-positive status was positively associated with anemia, a finding consistent with studies from Ethiopia [21]. The elevated risk may be explained by HIV-related bone marrow suppression, chronic inflammation, and increased susceptibility to opportunistic infections that interfere with red blood cell production and iron utilization.

## Conclusion

Anemia among women of reproductive age in Zambia persists as a significant public health concern, driven by a combination of biological, socio-demographic, and community-level factors. This study underscores the importance of multilevel and spatial approaches in revealing hidden geographic and structural disparities. Effective responses will require integrated interventions that move beyond individual supplementation to address broader systemic issues such as food insecurity, access to care, and environmental health risks. Future research should explore these unmeasured contextual drivers and evaluate the impact of locally tailored, multisectoral strategies.
